# A case series of early biologic therapy in guttate psoriasis: Targeting resident memory T cell activity as a potential novel therapeutic modality

**DOI:** 10.1016/j.jdcr.2022.04.019

**Published:** 2022-05-05

**Authors:** Akshay Flora, John W. Frew

**Affiliations:** a Department of Dermatology, Liverpool Hospital, Liverpool, Australia; b Faculty of Medicine, University of New South Wales, Sydney, Australia; c Laboratory of Translational Cutaneous Research, Ingham Institute of Applied Medical Research, Liverpool, Australia

**Keywords:** biologics, guttate psoriasis, IL-23, psoriasis vulgaris, resident memory T cells, GP, guttate psoriasis, IL, interleukin, PV, psoriasis vulgaris, T_RM_, resident memory T

## Introduction

Guttate psoriasis (GP) is a clinical subtype of psoriasis vulgaris (PV) manifesting in multiple erythematous plaques, predominantly on the trunk and limbs.^1^ Dysregulation of the T-helper 17 axis plays a central role in driving these inflammatory lesions through cytokines such as interleukin (IL) 17, IL-22, IL-23, and tumor necrosis factor-α.^2^ GP can be triggered by various infections, including viral illnesses (enteroviral diseases, COVID-19) streptococcal infection (pharyngitis and perineal streptococcal infection), as well as iatrogenic causes (use of β blockers, antimalarials).^3^^,^^4^ In the setting of infectious triggers, lesions most commonly develop 2 to 3 weeks after initial infection. A strong association has been found with HLA-Cw∗0602 allele positivity and recurrent infection, which may lead to recurrent outbreaks of GP.

GP will often spontaneously remit within 12 to 16 weeks upon onset, causing significant psychosocial distress while present. Current treatments for GP include treatment of the underlying infectious trigger (in the case of streptococcal infections), phototherapy, topical corticosteroids, and calcipotriol. Systemic immunosuppressive therapy and biologic therapy have been reported in GP but are less commonly used.^5^ Tonsillectomy has been promoted for recurrent GP triggered by streptococcal infection.

Approximately 25% to 39% of patients have been found to progress to chronic plaque psoriasis or PV after developing GP. One study for example, with a mean follow-up duration of 6.3 years, identified that 14 out of 36 patients (38.9%) progressed to PV.^6^ The pathogenic mechanism underlying this progression is incompletely understood but thought to involve resident memory T (T_RM_) cells.

Cutaneous T_RM_ cells are a unique subset of T cells in skin that express specific cellular markers, including CD69 and CD103.^6^ When stimulated by IL-23 via the IL23 receptor, they rapidly produce IL-17A, IL-17F, and IL22.^7^ They are linked to the cutaneous immunologic memory in various cutaneous disorders, such as PV, as well as recurrence of PV lesions at the sites of previous disease.^8^ Theoretically, T_RM_ cells may be involved in the progression of GP to PV through the development of T-helper 17 immunologic memory, leading to the chronic feed-forward inflammatory cascade characteristic of PV.^2^

We present a case series of 4 patients with clinically diagnosed GP with rapid and sustained clearance with IL-23 antagonism using risankizumab monotherapy. After 3 doses of risankizumab and withdrawal of therapy, no recurrence of disease was documented up to 24 months of follow-up. These cases support the possible role of T_RM_ cell antagonism in preventing the progression from GP to PV and a novel therapeutic approach for inducing rapid remission of GP.

## Case series

A summary of patient characteristics is presented in Table I. All patients were treated with risankizumab monotherapy with no adjuvant topical or physical therapies.

**Table I:** Demographic and clinical characteristics of patients included in this case series

Patient	Age	Gender	ASOT	FHx	Initial PASI score	Weeks until PASI100	Length of follow-up (psoriasis-free)
1	24	F	Positive	Nil	10.2	8	24 mo
2	37	M	Positive	Nil	8.8	4	18 mo
3	42	M	negative	Nil	11.4	12	12 mo
4	21	F	negative	Nil	12	4	24 mo

*ASOT,* Anti-streptolysin O titre; *FHx,* family history; *PASI,* psoriasis area and severity index.

**Fig 1:**
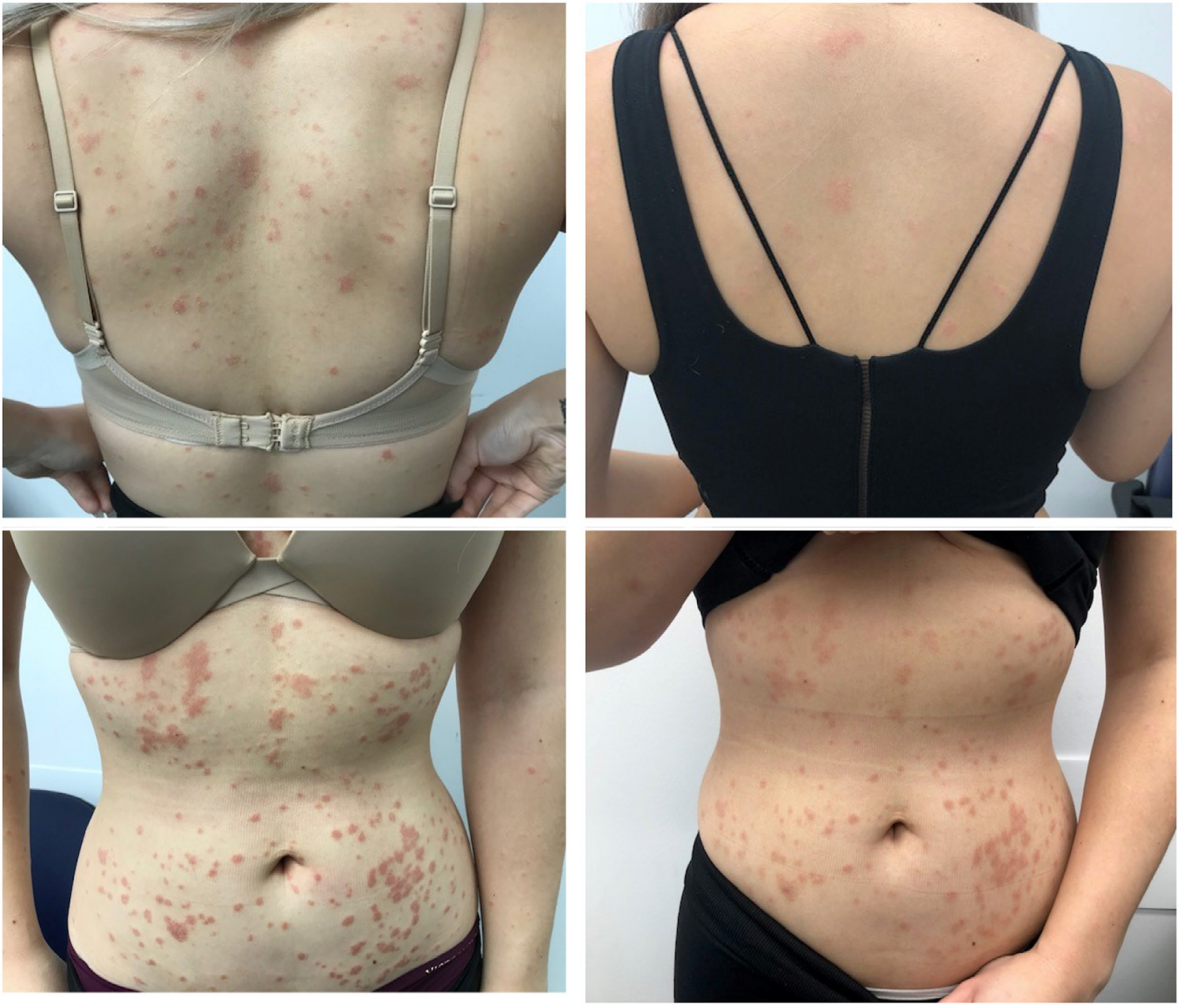
Clinical photographs of patient 1, demonstrating guttate psoriasis prior to the initiation of risankizumab therapy (*left panel*) and in week 4 (*right panel*). The patient achieved a psoriasis area and severity index score of 100 at week 8 of therapy.

Patient 1 is a 24-year-old woman who presented with an 8-week history of pink erythematous plaques on the trunk (Fig 1). She had a preceding pharyngitis approximately 2 weeks prior to disease onset. She had no other medical history and no regular medications. Serologic tests revealed a positive anti-streptolysin O titre, and negative HIV, hepatitis B, hepatitis C, and quantiferon serology. Risankizumab was commenced at 150 mg (2 injections of 75 mg/0.83 mL) at weeks 0, 4, and 16. By week 4, there was dramatic reduction in the thickness of plaques, with total resolution by week 8. Follow-up 24 months after resolution demonstrated no recurrence of GP.

**Fig 2:**
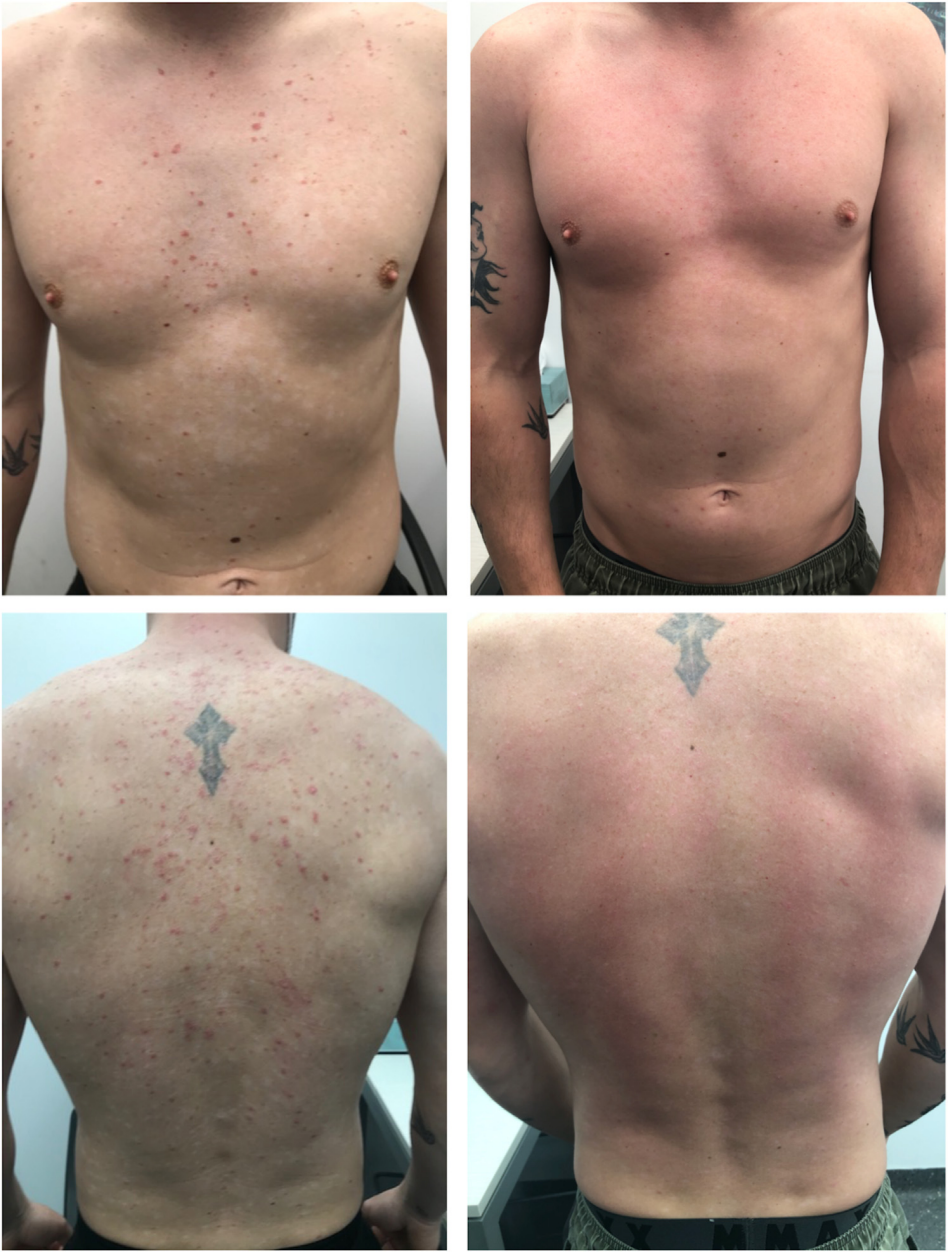
Clinical photographs of patient 2, demonstrating guttate psoriasis prior to the initiation of risankizumab therapy (*left panel*) and in week 4 (*right panel*). The patient achieved a psoriasis area and severity index score of 100 at week 4 of therapy.

Patient 2 is a 37-year-old man, who presented with a 3-week history of pruritic erythematous plaques on the anterior and posterior aspects of the chest (Fig 2). He had been diagnosed with COVID-19 4 weeks previously, with mild pharyngitis and no other symptoms. He had no other background medical history and no regular medications. Serology demonstrated negative HIV, hepatitis B, hepatitis C, and quantiferon tests. Risankizumab was commenced at 150 mg (2 injections of 75 mg/0.83 mL) at weeks 0, 4, and 16. By week 4, there was complete resolution of GP, and 18 months of follow-up demonstrated no recurrence.

**Fig 3:**
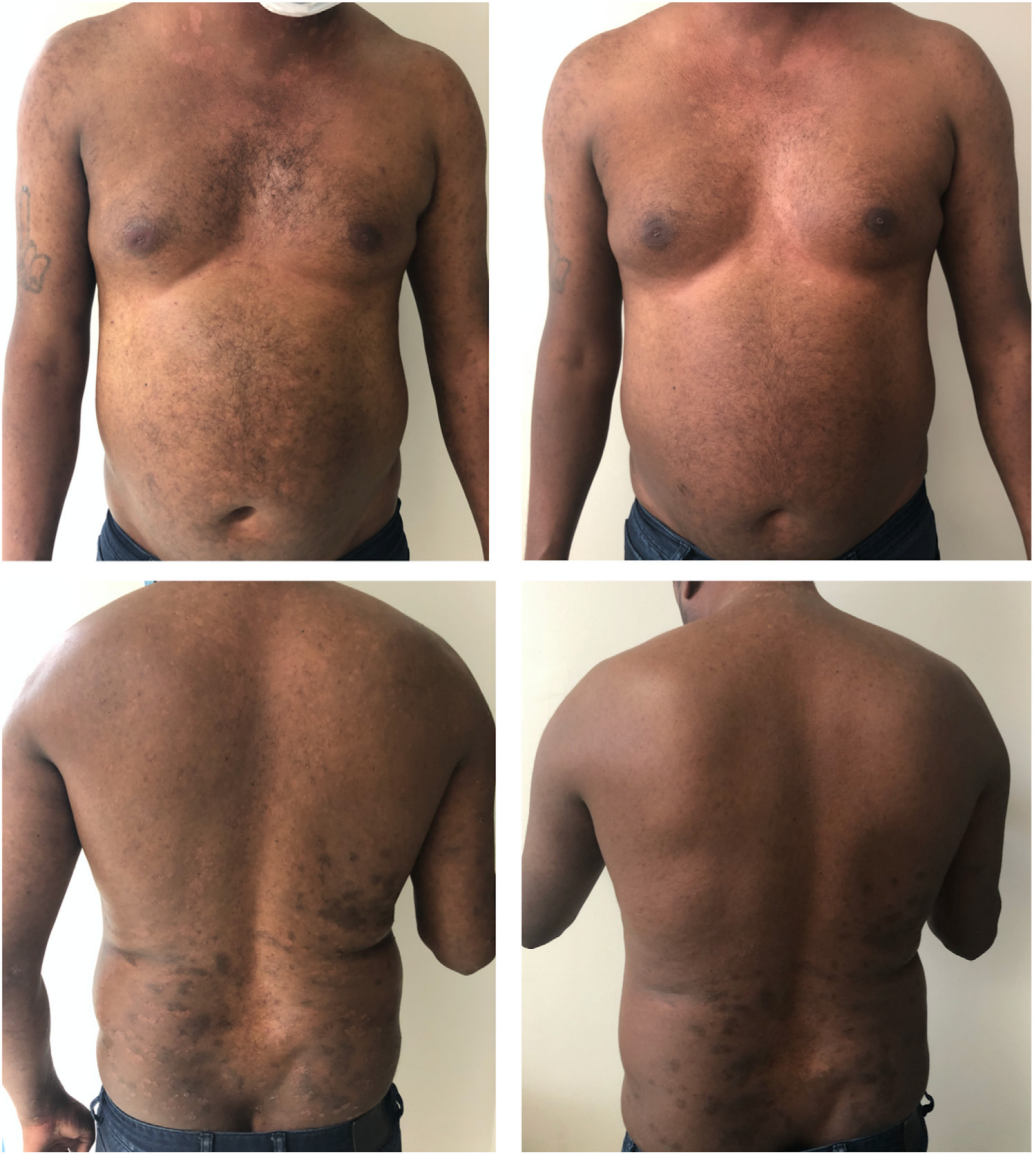
Clinical photographs of patient 3, demonstrating guttate psoriasis prior to the initiation of risankizumab therapy (*left panel*) and in week 4 (*right panel*). The patient achieved a psoriasis area and severity index score of 100 at week 12 of therapy.

Patient 3 is a 42-year-old man with a 2-year history of GP (Fig 3) with lack of response to topical corticosteroids, UV therapy, oral cyclosporine (3 mg/kg/day for 12 weeks), and acitretin (25 mg/day for 12 weeks). He had a background history of hyperlipidemia controlled with simvastatin 40 mg daily. Serology demonstrated negative HIV, hepatitis B, hepatitis C, and quantiferon tests. Risankizumab was commenced at 150 mg (2 injections of 75 mg/0.83 mL) at weeks 0, 4, and 16. Complete remission was seen at week 12, with no recurrence of lesions at 12 months after therapy.

**Fig 4:**
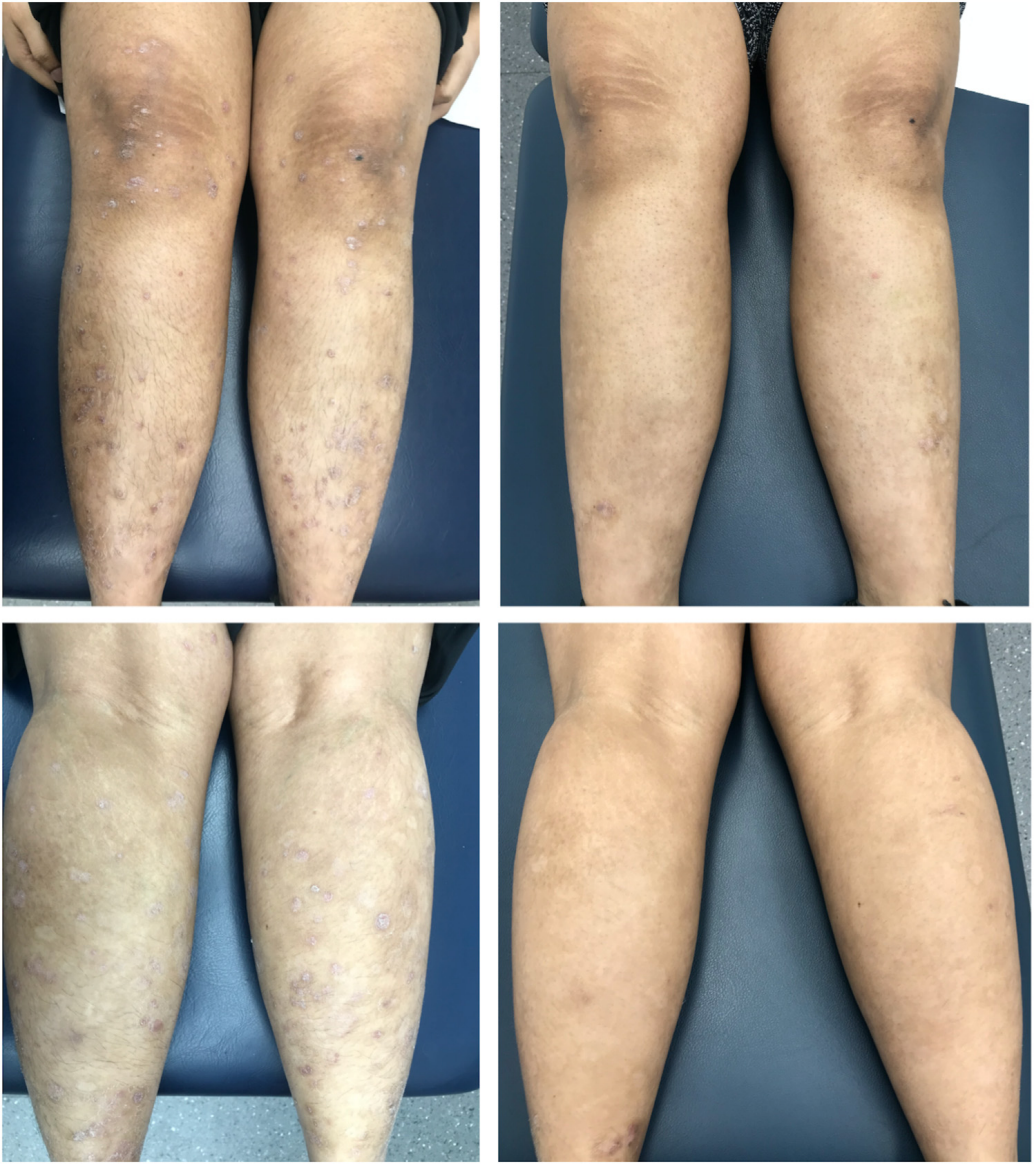
Clinical photographs of patient 4, demonstrating guttate psoriasis prior to initiation of risankizumab therapy (*left panel*) and in week 4 (*right panel*). The patient achieved a psoriasis area and severity index score of 100 at week 4 of therapy.

Patient 4 is a 21-year-old woman with a 6-week history of GP primarily on the trunk and lower portion of the limbs (Fig 4). This eruption occurred 3 weeks after an episode of pharyngitis. Her medical history was significant for obesity and insulin resistance, which was controlled with metformin 1000 mg daily. Serology demonstrated a positive anti-streptolysin O titre, and negative HIV, hepatitis B, hepatitis C, and quantiferon tests. Risankizumab was commenced at 150 mg (2 injections of 75 mg/0.83 mL) at weeks 0, 4, and 16. Complete clearance was observed by week 4, and no recurrence was noted 24 months after treatment cessation.

## Discussion

Although GP is a distinct variant of PV, it shares many pathophysiologic similarities to PV, including initial involvement of epidermal dendritic cells^8^ and high levels of IL-17 isoforms.^9^ Langerhans cell migration within the epidermis has been found to be impaired in both GP and PV, when compared with healthy controls.^9^ However, migration impairment was less in GP and PV, and patients with resolution of their GP demonstrated normalization of Langerhans cell migration in line with clinical resolution of disease.^9^ Therefore, from a pathophysiologic perspective, GP can be thought of as an innate immune stimulation of PV pathways without long-term perpetuation of feed-forward chronic inflammation.^2^ IL-23p19 antagonists are widely used in the treatment of PV, with significantly higher levels of clinical efficacy in terms of reaching a psoriasis area and severity index score of 75 and 90 than tumor necrosis factor-α inhibitors and some IL-17 inhibitors.^10^ Inhibition of IL-23p19 reduces activation and polarization of T-helper 17 cells, thereby reducing cytokines, including IL-17A, IL-17F, IL-22, and interferon-γ, which are linked to keratinocyte hyperproliferation and chronic feed-forward inflammation.^2^

Long-term remission with IL-23p19 antagonists such as guselkumab and risankizumab, has been demonstrated in PV for many months after therapy discontinuation, even after complete drug washout.^11^ Furthermore, patients who had a shorter duration of disease prior to commencing anti-IL23 treatments were identified to have a higher rate of sustained remission compared with those with long-standing disease.^12^ T_RM_ cells are proposed to explain these clinical findings, with both CD4^+^ and CD8^+^ T_RM_ cell populations upregulated in the epidermis of patients with PV.^13^ CD8^+^ epidermal T_RM_ cells are purportedly the most relevant to the pathogenesis of PV. Recent findings indicate that IL-23 is involved in the survival T_RM_ cells within cutaneous tissue of psoriasis patients, and that early IL-23 antagonism may reduce the formation and survival of T_RM_ cells.^13^ T_RM_ cells are also upregulated in skin where psoriasis was previously present, suggesting a resident immunologic ‘memory’ of disease. Our case series suggests a proof-of-concept that early treatment of GP with IL-23 antagonists can result in long-term remission. This has also been demonstrated in the setting of ustekinumab (an IL-12p40/IL-23p40 antagonist) therapy^5^ and guselkumab (IL-23p19 antagonist) therapy,^14^ supporting the use of other IL-23 antagonists in this setting. Short courses of treatment with IL-17 antagonists, such as secukinumab or ixekizumab, have also been demonstrated to contribute toward inducing remission of GP,^15^ which could be explained through blockade of IL-17 produced by T_RM_ cells.

The limitations of this study include the small number of patients in this case series, lack of an appropriate control group, and lack of follow-up past 24 months of treatment completion. Currently biologic therapies are not reimbursed for GP, and cost is a significant issue.

This case series presents a proof-of concept, and further research into potential cost-benefit of early intervention in GP would be required. As GP can spontaneously resolve without intervention, it is possible that the clinical resolution observed in this case series was due to the GP’s natural history, rather than due to IL-23 inhibition. Future studies require a placebo-controlled cohort and evaluation of T_RM_ cells in skin biopsies to determine the therapeutic potential of IL-23 antagonism in the setting of GP.

## Conflicts of interest

Dr Frew has conducted advisory work for Janssen, Boehringer-Ingelheim, Pfizer, Kyowa Kirin, LEO Pharma, Regeneron, Chemocentryx, AbbVie and UCB, participated in trials for Pfizer, UCB, Boehringer-Ingelheim, Eli Lilly, CSL, and received research support from Ortho Dermatologics and Sun Pharma. Dr Flora has no conflicts of interest to disclose.
